# Supplementary material for: The nasal methylome as a biomarker of asthma and airway inflammation in children
Source: Nat Commun. 2019 Jul 12;10:3095. doi: 10.1038/s41467-019-11058-3 (PMC6625976; doi:10.1038/s41467-019-11058-3)
Supplement: Supplementary file 1 — Supplementary Information [file 41467_2019_11058_MOESM1_ESM.pdf]

SUPPLEMENTARY INFORMATION

The Nasal Methylome as a Biomarker of Asthma and Airway Inflammation in Children

Cardenas et al.

Supplementary Figures

Supplementary Figure 1. Quantile-Quantile plots of expected *vs.* observed *p*-values from epigenome-wide association analyses (EWAS): *p*-values adjusted for confounders but no cell-type are in black and *p*-values from models further adjusted for cell-type heterogeneity using ReFACTor[4] (10 PCs) are in blue. Genomic inflation factors (λ) shown in plot for both analyses.

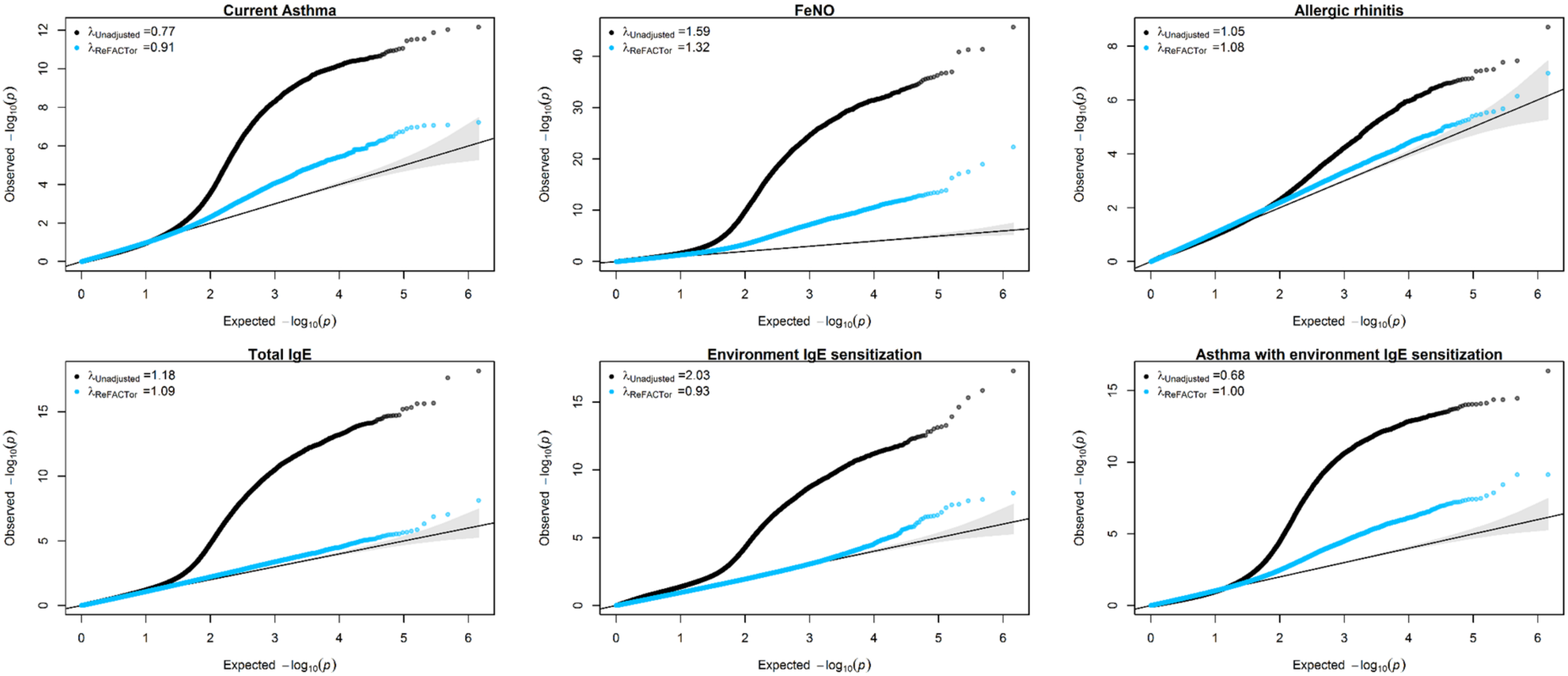

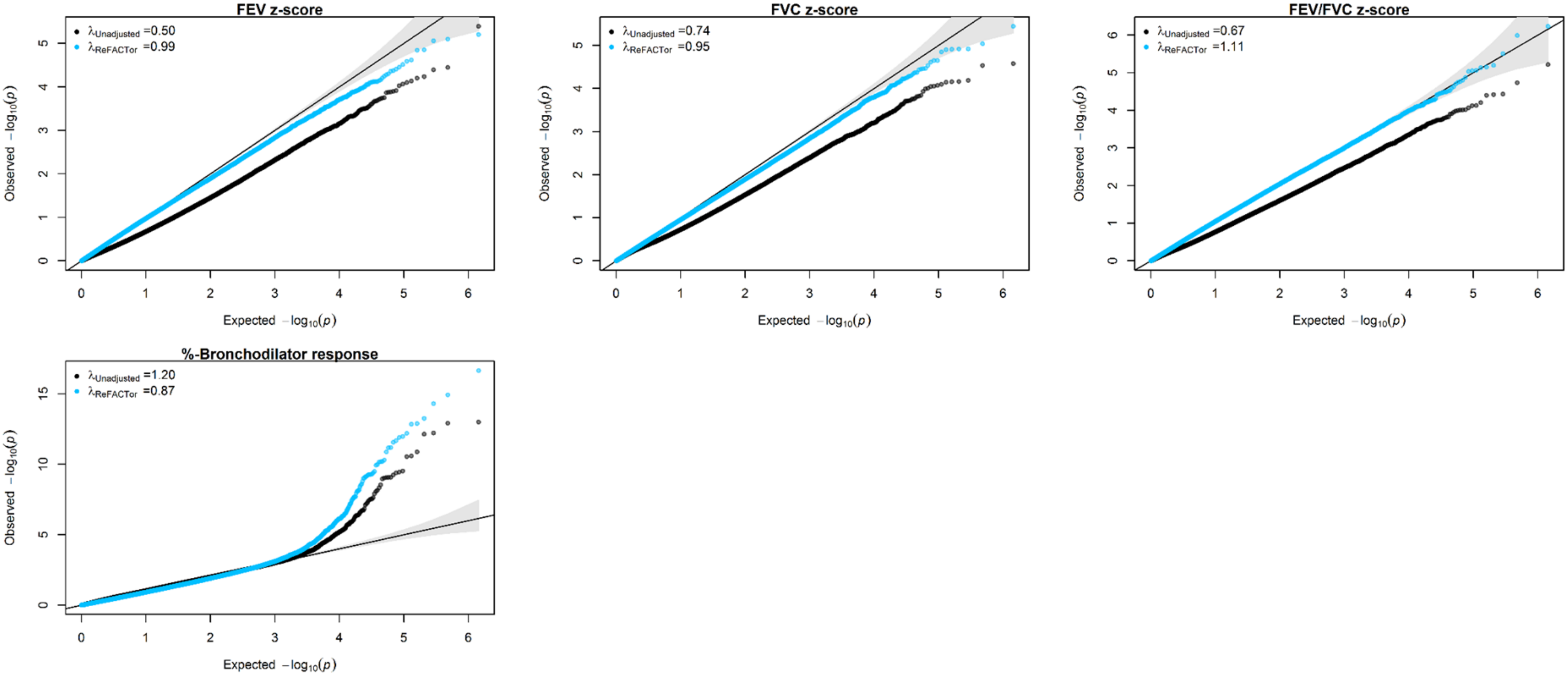
FEV z-score
λUnadjusted =0.50
λReFACTor =0.99
Observed −log10(p)
Expected −log10(p)
FVC z-score
λUnadjusted =0.74
λReFACTor =0.95
Observed −log10(p)
Expected −log10(p)
FEV/FVC z-score
λUnadjusted =0.67
λReFACTor =1.11
Observed −log10(p)
Expected −log10(p)
%-Bronchodilator response
λUnadjusted =1.20
λReFACTor =0.87
Observed −log10(p)
Expected −log10(p)

Supplementary Figure 2. Scatter-plot of chronological age and DNA methylation (DNAm-Age) estimated using the Horvath DNA methylation age calculator[5].

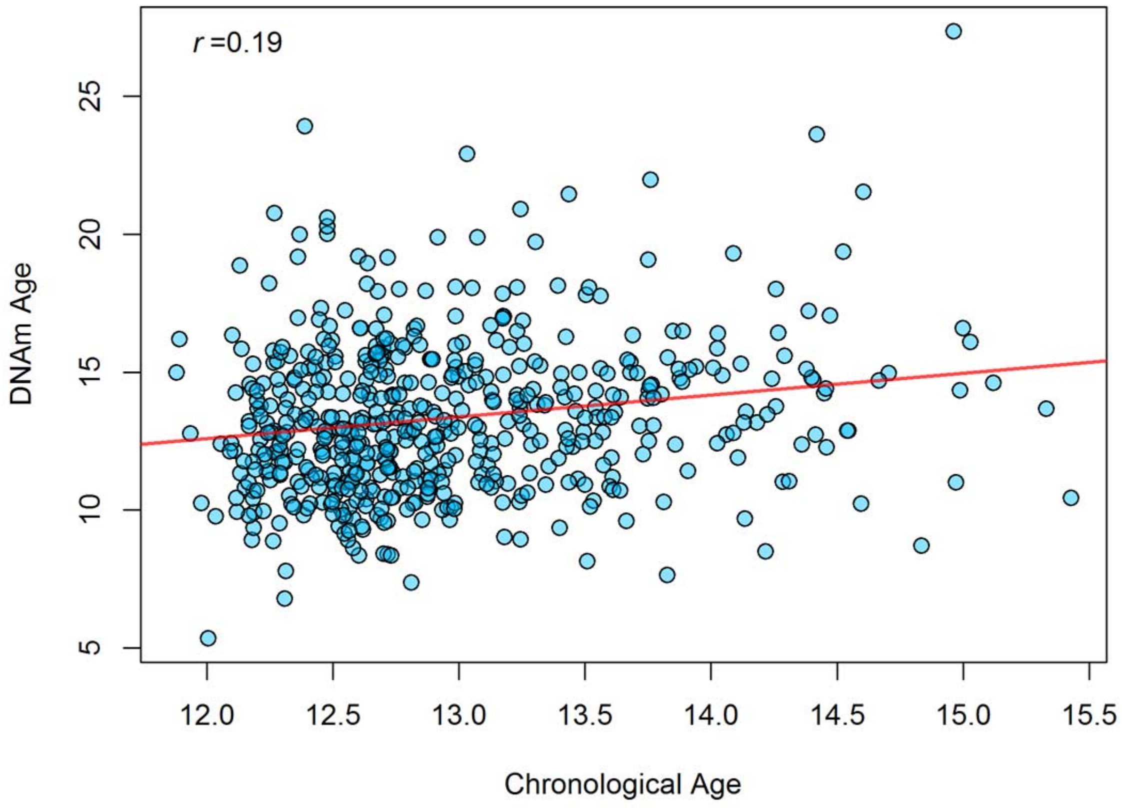

Supplementary Figure 3. Estimated principal components from ReFACTor[4] against eigen values.

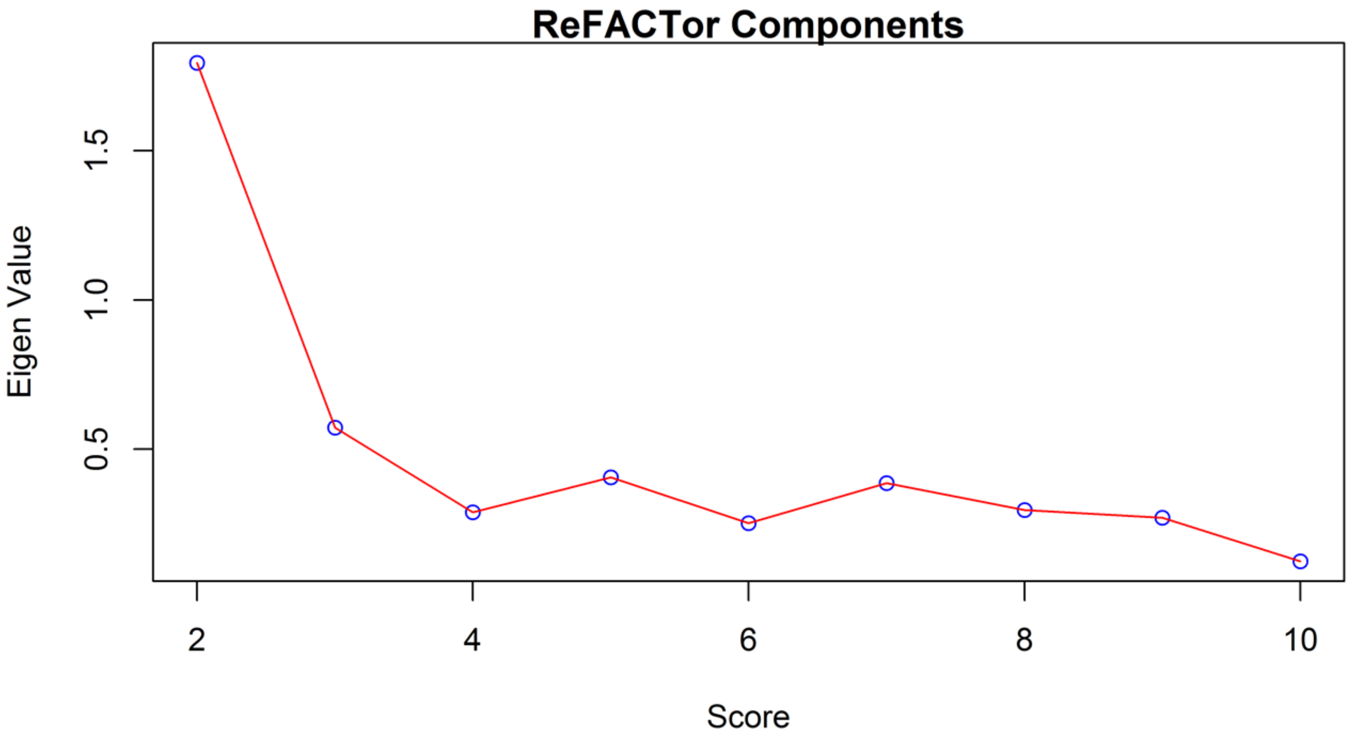

Supplementary Tables

Supplementary Table 1. Differentially methylated KEGG biological pathways among significant CpGs (FDR<0.05) found after adjustment for multiple pathways tested (FDR<0.10) using *missMethyl*.[1]

**KEGG Pathway**	**Genes**	**Allergic Asthma**			**Fractional Exhaled Nitric Oxide (FeNO)**		
		DMGs	**P*	FDR	DMGs	**P*	FDR
Asthma	26	5	1.76E-04	0.06	5	1.58E-05	5.17E-03
IL-17 signaling pathway	89	12	0.07	0.32	9	7.18E-05	0.01

KEGG= Kyoto Encyclopedia of Genes and Genomes

DMGs= Differentially methylated genes on pathway

**P*= unadjusted enrichment *p*-value

FDR=False Discovery Rate adjusted *p*-value for all pathways tested

Supplementary Table 2. Numbers and percent (%) overlap between differentially methylated CpGs (FDR<0.05) across different traits found in Epigenome-Wide Association Analyses (EWAS) for the nasal methylome.

EWAS	FeNO	Asthma	Total IgE	Environment IgE	Allergic Asthma	Bronchodilator Response (BDR)
**FeNO**<br>8,372 CpGs	---					
**Asthma**<br>285 CpGs	251<br>(88.1%)	---				
**Total IgE**<br>3 CpGs	3<br>(100%)	0	---			
**Environment IgE sensitization**<br>17 CpGs	16<br>(94.1%)	1<br>(5.9%)	3<br>(17.6%)	---		
**Allergic Asthma**<br>1,235 CpGs	979<br>(79.3%)	4<br>(0.3%)	2<br>(0.2%)	4 (0.3%)	---	
**Bronchodilator response (BDR)**<br>130 CpGs	0	0	0	0	0	---

*% indicate the proportion of overlap of each EWAS for each row with the respective EWAS on each column.

Supplementary Table 3. Summary results of Epigenome-Wide Associations for $\log_{10}$ total IgE concentrations.

**CpG**	**chr**	**position**	**% difference in DNAm**	**pvalue**	**qvalue**	**UCSC Gene Name**	**UCSC Gene Group**
cg16315329	3	50648706	-1.59	7.16E-09	0.004	*CISH;MAPKAPK3;CISH*	Body;TSS1500;5'UTR
cg06333800	7	151442481	3.59	1.31E-07	0.03	*PRKAG2;PRKAG2*	Body;Body
cg04983687	16	88558223	-3.26	8.72E-08	0.03	*ZFPM1*	Body

Supplementary Table 4. Summary results for Epigenome-Wide Associations of Environment IgE sensitization.

CpG	chr	position	% difference in DNAm	pvalue	qvalue	UCSC Gene Name	UCSC Gene Group
cg21220721	1	6341230	-3.56	2.86E-07	0.018	*ACOT7;ACOT7;ACOT7;ACOT7*	Body;Body;Body;Body
cg15006973	1	35258933	-1.64	6.04E-08	0.007	*GJA4*	5'UTR
cg16315329	3	50648706	-1.75	1.11E-06	0.047	*CISH;MAPKAPK3;CISH*	Body;TSS1500;5'UTR
cg22988305	3	52240549	-0.71	2.65E-07	0.018	*ALAS1;ALAS1;ALAS1;ALAS1*	Body;Body;Body;Body
cg16209444	3	58522771	1.53	4.50E-07	0.023	*ACOX2*	5'UTR
cg27378867	3	124519518	-1.78	2.93E-07	0.018	*ITGB5*	Body
cg08896592	5	106859843	-1.59	2.48E-07	0.018	*EFNA5*	Body
cg16911809	7	75402343	-2.39	2.18E-07	0.018	*CCL26*	5'UTR
cg06333800	7	151442481	4.95	1.94E-08	0.005	*PRKAG2;PRKAG2*	Body;Body
cg12445055	9	112229809	-1.78	3.75E-07	0.021	*PTPN3;PTPN3*	5'UTR;5'UTR
cg10408463	11	34638847	-1.40	3.38E-08	0.005	*EHF**	Upstream (3741 bps)
cg20372759	12	58162287	-1.69	8.09E-07	0.036	*CYP27B1*	TSS1500
cg04983687	16	88558223	-4.46	1.50E-08	0.005	*ZFPM1*	Body
cg19649900	19	1155030	2.81	3.78E-08	0.005	*SBNO2*	5'UTR
cg16606773	20	19955806	4.84	5.04E-09	0.004	*RIN2*	Body
cg12049875	20	19955868	2.44	7.25E-07	0.035	*RIN2*	Body
cg14536332	22	26077119	-1.44	1.35E-07	0.014	*ADRBK2*	Body

Supplementary Table 5. Associations between nasal DNA methylation and asthma in Project Viva among 14 CpG sites previously associated with asthma in a meta-analysis of blood DNA methylation of children[2].

**CpG**	**Chr**	**Gene**	**Meta-analysis coefficient[2]**	****%-difference in DNA methylation**	***P***	**¶%-difference in DNA methylation**	***P***
cg01445399	1	*LOC339524*	-0.0129	-5.34%	$9.98x10^{-10}$	-2.15%	$1.74x10^{-4}$
cg01770400	1	*SERPINC1*	-0.0087	-3.82%	$7.00x10^{-11}$	-1.80%	$5.38x10^{-6}$
cg16592897	1	*PROK1, KCNA10*	-0.0062	-1.85%	$4.87x10^{-7}$	-0.81%	$1.67x10^{-3}$
cg15344640	5	*MXD3, LMAN2*	-0.0095	-3.64%	$3.24x10^{-10}$	-1.69%	$2.48x10^{-5}$
cg11456013	6	*AMD1*	-0.0135	*NA*	*NA*	*NA*	*NA*
cg13628444	9	*RAPGEF1, MED27*	-0.0111	-4.98%	$1.36x10^{-9}$	-1.94%	$2.05x10^{-4}$
cg19764973	11	*STX3, MRPL16*	-0.0112	-2.79%	$1.86x10^{-8}$	-1.33%	$2.05x10^{-4}$
***cg08085199**	16	*CHTF8, CIRH1A*	-0.0117	-4.01%	$5.35x10^{-6}$	-1.38%	0.02
cg10142874	2	*TRIB2, LPIN1*	-0.0167	-4.51%	$1.09x10^{-9}$	-2.07%	$7.81x10^{-5}$
cg14011077	9	*PPP1R26, OLFM1*	-0.0148	-2.91%	$2.64x10^{-6}$	-1.23%	$3.06x10^{-3}$
cg13835688	9	*SLC25A25, PTGES2*	-0.0205	-5.99%	$1.57x10^{-9}$	-2.49%	$1.66x10^{-4}$
cg03131767	12	*VPS37B, ABCB9*	-0.0148	-4.41%	$2.47x10^{-6}$	-1.62%	$1.13x10^{-3}$
cg01901579	14	*DICER1, CLMN*	-0.0183	-5.67%	$2.10x10^{-9}$	-2.15%	$5.89x10^{-4}$
***cg06483820**	17	*MSI2, AKAP1*	-0.0139	-3.85%	$1.73x10^{-4}$	-1.11%	0.06

**Adjusted for sex, race, age at sample collection, BMI *z-score*, maternal education, smokers living in the house, season as well as sine and cosine of season of sample collection.

¶Additionally adjusting for 10 PCs as surrogate of cell-type variation from ReFACTor

NA: Not measured by Illumina EPIC BeadChip but measured in 450K.

*Excluded from main analyses for this manuscript because of the presence of a SNP at the probe: **cg08085199** has a SNP (rs3815821) with MAF=0.077 while **cg06483820** has a SNP (rs72843421) with MAF=0.108. However, we ran models to include these sites for the replication table.

Supplementary Table 6. Summary results of replication analyses in nasal epithelial cells and environment IgE sensitization.

				Cardenas et al. Nasal 850K Data			Forno et al. Nasal 450K Data[3]		
**CpG**	**chr**	**position**	**UCSC Gene Name**	**% difference in DNAm**	**pvalue**	**qval**	**Coefficient (M-value)**	**pvalue**	**qval**
cg21220721	1	6341230	*ACOT7*	-3.56	2.86E-07	0.018			
cg15006973	1	35258933	*GJA4*	-1.64	6.04E-08	0.007	-2.30	9.56E-26	3.35E-25
cg16315329	3	50648706	*CISH;MAPKAPK3*	-1.75	1.11E-06	0.047			
cg22988305	3	52240549	*ALAS1*	-0.71	2.65E-07	0.018			
cg16209444	3	58522771	*ACOX2*	1.53	4.50E-07	0.023	3.10	4.32E-15	1.01E-14
cg27378867	3	124519518	*ITGB5*	-1.78	2.93E-07	0.018			
cg08896592	5	106859843	*EFNA5*	-1.59	2.48E-07	0.018			
cg16911809	7	75402343	*CCL26*	-2.39	2.18E-07	0.018			
cg06333800	7	151442481	*PRKAG2*	4.95	1.94E-08	0.005	0.94	4.59E-08	8.03E-08
cg12445055	9	112229809	*PTPN3*	-1.78	3.75E-07	0.021			
cg10408463	11	34638847		-1.4	3.38E-08	0.005			
cg20372759	12	58162287	*CYP27B1*	-1.69	8.09E-07	0.036	-1.50	9.59E-28	6.71E-27
cg04983687	16	88558223	*ZFPM1*	-4.46	1.50E-08	0.005			
cg19649900	19	1155030	*SBNO2*	2.81	3.78E-08	0.005	0.03	0.798	0.841
cg16606773	20	19955806	*RIN2*	4.84	5.04E-09	0.004	0.09	0.345	0.483
cg12049875	20	19955868	*RIN2*	2.44	7.25E-07	0.035	0.03	0.841	0.841
cg14536332	22	26077119	*ADRBK2*	-1.44	1.35E-07	0.014			

Blank cells; not available in data from Forno *et al*[3].

Supplementary Table 7. Quality control and sequential exclusion of probes for analyses.

Quality control step Description	Number of probes excluded	Total
Initial total number of probes		866,836
Failed detection *p*-value (*P*>0.05) for ≥5% of samples	4,161	
Probes on X and Y chromosomes	18,978	
Non CpG probes (“ch” and “rs”)	2,835	
SNP at the single base extension (MAF≥5%)	5,516	
SNP at the CpG site (MAF≥5%)	5,215	
SNP within the probe (MAF≥5%)	70,737	
Previously identified cross-reactive probes (initially 43,254)	40,319	
Final number of high quality probes		719,075

Supplementary References

1. Phipson B, Maksimovic J, Oshlack A. missMethyl: an R package for analyzing data from Illumina's HumanMethylation450 platform. *Bioinformatics* **32**, 286-288 (2015).

2. Xu C-J*, et al.* DNA methylation in childhood asthma: an epigenome-wide meta-analysis. *The lancet Respiratory medicine* **6**, 379-388 (2018).

3. Forno E*, et al.* DNA methylation in nasal epithelium, atopy, and atopic asthma in children: a genome-wide study. *The Lancet Respiratory Medicine* **7**, 336-346 (2019).

4. Rahmani E*, et al.* Sparse PCA corrects for cell type heterogeneity in epigenome-wide association studies. *Nature methods* **13**, 443 (2016).

5. Horvath S. DNA methylation age of human tissues and cell types. *Genome biology* **14**, 3156 (2013).
