# Supplementary material for: The nasal methylome as a biomarker of asthma and airway inflammation in children
Source: Nat Commun. 2019 Jul 12;10:3095. doi: 10.1038/s41467-019-11058-3 (PMC6625976; doi:10.1038/s41467-019-11058-3)
Supplement: Supplementary file 3 — Reporting Summary [file 41467_2019_11058_MOESM3_ESM.pdf]

nature research

Corresponding author(s): Andres Cardenas

Last updated by author(s): May 23, 2019

Reporting Summary

Nature Research wishes to improve the reproducibility of the work that we publish. This form provides structure for consistency and transparency in reporting. For further information on Nature Research policies, see Authors & Referees and the Editorial Policy Checklist.

Statistics

For all statistical analyses, confirm that the following items are present in the figure legend, table legend, main text, or Methods section.

n/a	Confirmed	
☐	☒	The exact sample size (n) for each experimental group/condition, given as a discrete number and unit of measurement
☐	☒	A statement on whether measurements were taken from distinct samples or whether the same sample was measured repeatedly
☐	☒	The statistical test(s) used AND whether they are one- or two-sided<br>*Only common tests should be described solely by name; describe more complex techniques in the Methods section.*
☐	☒	A description of all covariates tested
☐	☒	A description of any assumptions or corrections, such as tests of normality and adjustment for multiple comparisons
☐	☒	A full description of the statistical parameters including central tendency (e.g. means) or other basic estimates (e.g. regression coefficient) AND variation (e.g. standard deviation) or associated estimates of uncertainty (e.g. confidence intervals)
☐	☒	For null hypothesis testing, the test statistic (e.g. F, t, r) with confidence intervals, effect sizes, degrees of freedom and P value noted<br>*Give P values as exact values whenever suitable.*
☒	☐	For Bayesian analysis, information on the choice of priors and Markov chain Monte Carlo settings
☒	☐	For hierarchical and complex designs, identification of the appropriate level for tests and full reporting of outcomes
☐	☒	Estimates of effect sizes (e.g. Cohen's d, Pearson's r), indicating how they were calculated

Our web collection on statistics for biologists contains articles on many of the points above.

Software and code

Policy information about availability of computer code

Data collection	We used epigenetic age acceleration from the residuals of a linear model regressing DNAm-Age on chronological age as reported in the Horvath's online calculator: (https://dnamage.genetics.ucla.edu/. All statistical analyses were carried out using R, version 3.5.0 (www.r-project.org/).
Data analysis	Among 547 participants with high quality DNAm data eligible for analyses we report our sample's demographic and biological characteristics using means, standard deviations, or proportions. We performed epigenome-wide association analyses (EWAS) CpG-by-CpG by fitting linear regression models using limma with moderate test-statistics using an empirical Bayes estimation. In EWAS models we adjusted for variables selected a priori and based on PCA plots: child race/ethnicity, sex, age at sample collection in days, age and sex-specific BMI z-score using US national reference data, smokers currently living in the house, sine and cosine of season of sample collection, maternal education in pregnancy and cell-type heterogeneity. We performed 10 independent EWAS to analyze nasal DNAm in relation to: 1) current asthma vs. never 2) current allergic asthma vs. never 3) FeNO 4) total serum IgE levels 5) any environment specific IgE 6) FEV z-score 7) FVC z-score 8) FEV/FVC z-score 9) Bronchodilator response (BDR) 10) current allergic rhinitis vs. never. We bioinformatically controlled for cell-type composition using ReFACTor, a reference-free method to adjust for cell-type composition in genome-wide DNAm studies from heterogenous tissues53. We chose ReFACTor as it has been shown to control the false positive rate even when compared to reference-based methods37. We adjusted for the first 10 principal components from ReFACTor (Supplementary Figure S3) as proxy for nasal cellular heterogeneity. CpG-by-CpG EWAS results were adjusted for multiple comparisons using a Bonferroni correction ($P<6.95x10^{-8}$). Quantile-quantile plots for the regression P-values were used to visually inspect genomic inflation and we report the genomic inflation factor (λ) for unadjusted and cell-type adjusted analyses. We performed regional DNAm analyses using DMRcate54 to identify differentially methylated regions (DMRs) associated with each trait. We defined a significant DMR as a DNAm region ≥5-CpGs and evaluated statistical significance using a Stouffer FDR adjusted P<0.05. We evaluated the overlap among differentially methylated CpGs for each EWAS surviving multiple testing adjustment.

For manuscripts utilizing custom algorithms or software that are central to the research but not yet described in published literature, software must be made available to editors/reviewers. We strongly encourage code deposition in a community repository (e.g. GitHub). See the Nature Research guidelines for submitting code & software for further information.

Data

Policy information about availability of data

All manuscripts must include a data availability statement. This statement should provide the following information, where applicable:

- Accession codes, unique identifiers, or web links for publicly available datasets
- A list of figures that have associated raw data
- A description of any restrictions on data availability

Data and Availability: Datasets generated and analyzed during the current study are not publicly available because we did not obtain consent for such public release of epigenetic data from participants. However, data to generate figures and tables are available from the corresponding author with the appropriate permission from the Project Viva study team and investigators (project_viva@hphc.org) upon reasonable request. Summary statistics for all EWAS performed can be requested by investigator by contacting the Project Viva team (project_viva@hphc.org) or corresponding author (andres.cardenas@berkeley.edu)

Field-specific reporting

Please select the one below that is the best fit for your research. If you are not sure, read the appropriate sections before making your selection.

☒ Life sciences ☐ Behavioural & social sciences ☐ Ecological, evolutionary & environmental sciences

For a reference copy of the document with all sections, see nature.com/documents/nr-reporting-summary-flat.pdf

Life sciences study design

All studies must disclose on these points even when the disclosure is negative.

Sample size	Children were participants in Project Viva, a prospective pre-birth cohort study recruited between 1999 and 2002 during the mothers' first prenatal visits at Atrius Harvard Vanguard Medical Associates, a multispecialty medical group practice in Massachusetts, United States. Eligibility criteria included fluency in English, gestational age less than 22 weeks at the first prenatal visit, and singleton pregnancy. Of the total 2,128 live births, 547 children were re-contacted during an early-teen in-person visit (mean 12.9y) and provided consent for nasal swab sample collection.
Data exclusions	We first performed quality control at the sample level, excluding samples with overall low intensities which indicates low quality (intensities <10.5; n=3), samples that mismatched on recorded sex (n=4) and samples with mixed genotype distributions on the measured SNP probes (59 SNP probes) indicating possible sample contamination (n=8). In addition, we excluded technical duplicates (n=35). A total of 547 high quality samples were retained for analyses.
Replication	Replication in Epithelial Nasal Cells. We sought to replicate our top differentially methylated findings of asthma and allergic asthma in an external cohort with nasal epithelial cells collected from the posterior portion of the inferior turbinate from the Inner City Asthma Consortium. Briefly, in this study samples of nasal epithelial cells from 36 atopic asthmatics and 36 controls with at least 80% ciliated epithelial cells were collected and DNA methylation was measured using Illumina's Infinium Human Methylation 450K BeadChip. We downloaded publicly available data from the Gene Expression Omnibus repository (GSE65163) 6. To allow for direct comparability we carried out the same pre-processing and analytical strategy used in our study, including adjusting for cell-type using ReFACTor (9 PCs). Among differentially methylated CpGs found for asthma and allergic asthma we compared differences in DNA methylation in adjusted models and controlling the FDR<0.05. We further tested for replication using a second independent study from The Epigenetic Variation and Childhood Asthma in Puerto Ricans (EVA-PR), a case-control study of childhood asthma in Puerto Rico. Briefly, in this study, nasal epithelial samples from 483 participants aged 9-20 years were collected, and DNA methylation was measured using the Illumina's Infinium Human Methylation 450K BeadChip. Atopy was defined as at least one positive IgE to five common aeroallergens in Puerto Rico; asthma was defined as physician's diagnosis plus at least one episode of wheezing in the previous year. We used data from the EVA-PR EWAS for atopic asthma (vs non-atopic controls) as replication for our analyses on asthma and atopic asthma; and the EVA-PR EWAS for atopy as replication for our analysis of IgE sensitization. We adjusted the FDR<0.05 among CpGs found in both analyses.
Randomization	Sample plates and chips were randomized to ensure balance by sex, current asthma status, current allergic rhinitis, and race to minimize potential confounding by batch effects.
Blinding	No blinding as this was an observational human study.

Reporting for specific materials, systems and methods

We require information from authors about some types of materials, experimental systems and methods used in many studies. Here, indicate whether each material, system or method listed is relevant to your study. If you are not sure if a list item applies to your research, read the appropriate section before selecting a response.

Materials & experimental systems

n/a	Involved in the study
☒	☐ Antibodies
☒	☐ Eukaryotic cell lines
☒	☐ Palaeontology
☒	☐ Animals and other organisms
☐	☒ Human research participants
☒	☐ Clinical data

Methods

n/a	Involved in the study
☒	☐ ChIP-seq
☒	☐ Flow cytometry
☒	☐ MRI-based neuroimaging

Human research participants

Policy information about studies involving human research participants

Population characteristics	Study Characteristics. We collected nasal DNA samples year-round: 33.6% in summer, 21.9% in fall, 19% in winter and 25.4% in spring. We measured nasal DNAm among N=547 Project Viva participants at the early teen visit with a mean age 12.9y (SD=0.65), range 11.9-15.3y. Participants were 50.6% male, and 67.1% White, 16.1% Black, 4.2 % Hispanic, 3.1% Asian and 9.3% of more than one race. Overall, 12% reported current asthma and 16.7% reported symptoms consistent with current allergic rhinitis. Of the 366 participants with IgE sensitization testing, 58.7% were sensitized to environmental allergens (Table 1).
Recruitment	Study Population. Children were participants in Project Viva, a prospective pre-birth cohort study recruited between 1999 and 2002 during the mothers' first prenatal visits at Atrius Harvard Vanguard Medical Associates, a multispecialty medical group practice in Massachusetts, United States. Eligibility criteria included fluency in English, gestational age less than 22 weeks at the first prenatal visit, and singleton pregnancy. Of the total 2,128 live births, 547 children were re-contacted during an early-teen in-person visit (mean 12.9y) and provided consent for nasal swab sample collection. Mothers provided written informed consent at recruitment and at postpartum follow-up visits.
Ethics oversight	The Institutional Review Board of Harvard Pilgrim Health Care reviewed and approved all study protocols.

Note that full information on the approval of the study protocol must also be provided in the manuscript.
