# Supplementary material for: The nasal methylome as a biomarker of asthma and airway inflammation in children
Source: Nat Commun. 2019 Jul 12;10:3095. doi: 10.1038/s41467-019-11058-3 (PMC6625976; doi:10.1038/s41467-019-11058-3)
Supplement: Supplementary file 4 — Description of Additional Supplementary Files [file 41467_2019_11058_MOESM4_ESM.pdf]

Description of Additional Supplementary Files

File Name: Supplementary Data 1
Description: Summary results of Epigenome-Wide Association Analyses of current asthma for CpG sites found to be differentially methylated (FDR<0.05).

File Name: Supplementary Data 2
Description: Summary results of Epigenome-Wide Association Analyses of fractional exhaled nitric oxide (FeNO) for CpG sites found to be differentially methylated (FDR<0.05).

File Name: Supplementary Data 3
Description: Summary results of Epigenome-Wide Association Analyses of allergic asthma for CpG sites found to be differentially methylated (FDR<0.05).

File Name: Supplementary Data 4
Description: Summary results of Epigenome-Wide Association Analyses of Bronchodilator Response (BDR) for CpG sites found to be differentially methylated (FDR<0.05).

File Name: Supplementary Data 5
Description: Complete list of differentially methylated regions (DMRs) ranked by lowest FDR P-value associated with $\log_{10}$ FeNO.

File Name: Supplementary Data 6
Description: Summary results of replication analyses in nasal epithelial cells and current asthma.

File Name: Supplementary Data 7
Description: Summary results of replication analyses in nasal epithelial cells and allergic asthma.
